# A *Bacillus pretiosus* Biofertilizer Enhances Early Growth, Nutritional Profile, and Rhizospheric Dynamics in *Quercus* Species for Forest Restoration

**DOI:** 10.3390/life16091558

**Published:** 2026-09-17

**Authors:** Marina Robas-Mora, Daniel González-Reguero, Vanesa M. Fernández-Pastrana, Diana Penalba-Iglesias, Agustín Probanza, Pedro A. Jiménez-Gómez

**Affiliations:** Department of Pharmaceutical and Health Sciences, Universidad San Pablo-CEU, CEU Universities, Urbanización Montepríncipe, 28660 Boadilla del Monte, Spain; marina.robasmora@ceu.es (M.R.-M.); diana.penalbaiglesias@ceu.es (D.P.-I.); a.probanza@ceu.es (A.P.); pedro.jimenezgomez@ceu.es (P.A.J.-G.)

**Keywords:** *Bacillus pretiosus*, rhizospheric microbiota, nitrogen metabolism, biofertilization, forest restoration

## Abstract

Plant growth-promoting bacteria (PGPBs) have emerged as essential biological inputs to improve the establishment, physiological performance and survival of forest species used in ecological restoration programs. The decline of *Quercus* forests throughout the Mediterranean basin requires innovative and sustainable strategies to improve early seedling establishment. Among possible PGBs, *Bacillus pretiosus* has attracted significant interest due to its ability to promote plant growth, modulate the nutrient status of the host, and safely interact with native rhizosphere microbiota. At the same time, evaluating urban wastewater waste using PGPB offers a promising circular economy approach. To provide an integrated functional and ecological assessment of *B. pretiosus* C1, this in silico study harmonizes and reinterprets experimental datasets through contrasting fertigation regimes—water (W), wastewater treatment plant effluent (EDAR) and electrochemically treated effluent (EDARST)—evaluating inoculation (C1) versus uninoculated controls (C0). Inoculation resulted in a consistent positive biometric response, significantly promoting outbreak length (up to +16.2% in EDARST). In addition, *B. pretiosus* C1 improved nitrogen leaf metabolism, raising crude protein levels (+11.8%), soluble protein and total amino acids (+15.4%). Sequencing and functional profiling of the high-yield 16S rRNA gene revealed that the introduction of strains did not significantly alter the alpha-diversity or overall beta structure of the native rhizosphere microbiome, demonstrating high ecological compatibility. Phenotypic profiling of the resistome by cenoantibiogram did not confirm an increase in resistance to antibiotics at the community level, instead showing a significant reduction in the minimum inhibitory concentration (MIC) for imipenem and amoxicillin/clavulanic acid under the fertigation of effluents. Together, these findings demonstrate that combining effluents valued with *p* > 0.05 *B. pretiosus* C1 shows a consistent functional profile with a high-value biotechnological agent, improving the biometric and nutritional status of seedlings without altering the stability of the soil microbial community. This supports its potential application as a safe biofertilizer candidate within forest restoration strategies and One Health, although more prospective field trials will be needed to validate each site.

## 1. Introduction

Mediterranean forest ecosystems dominated by species of the genus *Quercus* play a fundamental role in Mediterranean ecosystems, contributing to soil stability, maintenance of biodiversity and regulation of key ecological processes [1,2]. However, these species’ natural regeneration is limited by the high recurrence of environmental disturbances, which affect both the vegetation cover and the structure, functionality and biodiversity of the soil [3,4,5].

Conventional reforestation strategies often focus on biometric parameters or seedling survival, often using chemical fertilizers. However, these approaches have important limitations, as they do not reproduce the complex biological interactions of the soil, can generate soil imbalances and do not consider the role of the microbiota in plant nutrition, stress tolerance and ecosystem stability [6,7]. Among the strategies explored to promote soil restoration is the use of recovered organic matrices, including treated waste from wastewater treatment plants (WWTPs), capable of acting as a support for beneficial microorganisms and as a source of nutrients for the plant and soil microbiota [8,9,10].

The incorporation of plant growth-promoting bacteria (PGPB) represents a further advance in this approach. Strains belonging to genera such as *Bacillus* spp. and *Pseudomonas* spp. have exhibited an improvement in plant nutrition, a modulation of plant metabolism and an increase in tolerance to abiotic stresses [11,12,13]. Within this group, the species of the genus *Bacillus* stand out for their metabolic plasticity, capacity for persistence in the soil and potential to modulate processes associated with plant growth and rhizospheric dynamics [14]. In particular, *Bacillus pretiosus* (*B. pretiosus*) has emerged as a strain of interest for its possible involvement in nutrient acquisition, tolerance to abiotic stress, and functional modulation of the soil microbiome [15,16,17].

Despite the growing interest in PGPBs in plant restoration, important gaps remain regarding the consistency of their effects between different forest species, the extrapolation of controlled conditions to the field and their interaction with the native microbiota. Beyond the direct effect on growth, the introduction of PGPB in forest systems raises questions about its functional persistence at different stages of development and its influence on ecologically relevant processes such as nitrogen dynamics, structural metabolism or selective pressure on antibiotic resistance in the soil [18,19].

The functional characterization of *B. pretiosus* requires multidimensional approaches that integrate the physiological response of the plant, the mineral nutritional composition, including (P) phosphorus, (K) potassium, (Ca) calcium, and (Mg) magnesium [20], the taxonomic evaluation of the rhizospheric community by sequencing the *16S rRNA* gene [21,22] and the analysis of the community resistome by a cenoantibiogram [23,24]. The cross-sectional combination of datasets obtained under different contexts allows the identification of robust functional patterns and the evaluation of the ecological stability of the strain-induced response.

Due to the complexity of plant–rhizosphere interactions, the functional evaluation of PGPB requires approaches capable of integrating different experimental models, plant species, and environmental conditions. The cross-sectional combination of datasets obtained under different contexts allows the identification of robust functional patterns and the evaluation of the ecological stability of the strain-induced response.

The aim of this study was to provide an integrated evaluation of the functional and ecological profile of *B. pretiosus* as a plant growth-promoting bacterium (PGPB) in forest species of the genus *Quercus*. To achieve this, data from previous experimental studies were harmonized and integrated, considering only variables that were biologically and methodologically comparable. Specifically, the study aimed to: (i) assess the effects of *B. pretiosus* inoculation on plant growth-related traits, including shoot length, shoot biomass and other available biometric parameters; (ii) evaluate its influence on plant nutritional status, with particular emphasis on nitrogen-related variables such as crude protein, soluble protein and total amino acid contents; (iii) determine whether inoculation was associated with changes in the functional and taxonomic diversity of the rhizospheric microbiota, using indicators derived from Biolog EcoPlates^®^ and 16S rRNA gene sequencing; (iv) assess its effects on the community-level phenotypic antibiotic resistance profile of the rhizospheric microbiota based on minimum inhibitory concentration (MIC) values obtained through community antibiogram analyses; and (v) develop an integrated functional synthesis to identify the most consistent traits associated with *B. pretiosus* and evaluate its potential for application in biofertilization and forest restoration strategies.

## 2. Materials and Methods

### 2.1. Cross-Sectional Experimental Approach

The present paper was proposed as a cross-sectional, cross-study secondary-data analysis based on the integration and reinterpretation of data from previous experimental studies developed by the research group on forest species of the genus *Quercus*. The source studies covered different *Quercus* species, experimental conditions and stages of plant development, but shared a common experimental structure: the comparison between treatments without bacterial inoculation and treatments inoculated with *B. pretiosus*, in combination with different fertigation matrices. This structure allowed for cross-sectional integration aimed at identifying consistent functional patterns associated with the strain.

For the present analysis, only those variables that were available and comparable between studies from a biological and methodological point of view were selected. The integrated variables were grouped into different functional domains: plant biometric response, nutritional composition, functional activity of the rhizospheric microbiota, taxonomic structure of the bacterial community and community profile of antibiotic resistance. The interpretation of the results was carried out in a comparative manner, avoiding considering the source studies as a single homogeneous experiment.

### 2.2. PGPB Strain Subject to Cross-Sectional Analysis: Bacillus pretiosus

The strain subject to this cross-sectional analysis was *B. pretiosus* C1 (CECT 30674^T^/DSM 114702^T^), whose taxonomic identity and biosafety profile were previously established via whole-genome sequencing (GenBank: JAOXJG000000000) and functional characterization by Robas Mora et al. (2023) [15] as a plant growth-promoting bacterium of biotechnological interest. The strain belongs to the collection of the Environmental Microbial Biotechnology (MICROAMB) research group at the CEU San Pablo University. To contextualize its selection as the focus of this paper, Table 1 shows its main identification data, PGPB traits and biosafety criteria described in the source studies.

The selection of *B. pretiosus* as the focus of the analysis was based on the combination of two criteria: its functional potential as a PGPB and its biosafety profile. In the present paper, the data from the source studies were reorganized to compare, whenever possible, the treatments inoculated with this strain versus their non-inoculated controls within each fertigation matrix.

### 2.3. Source Studies and Experimental Models Included

The data came from previous experimental studies published or in the process of being published by the research group. Each source study was considered as an independent documentary and experimental unit, and their data were extracted, harmonized and reorganized for the present cross-sectional analysis. These studies were selected because they share a comparable experimental structure based on the evaluation of fertigation matrices, with or without inoculation with *B. pretiosus*, and because they included variables related to plant and rhizospheric response.

Three types of experimental models were integrated. The first corresponded to a germination and initial development trial of *Quercus ilex* from acorns, carried out under controlled laboratory conditions. The second included growth trials of one-year-old seedlings of *Q. ilex*, *Q. pyrenaica* and *Q. suber*, grown to complete the second year of development under controlled conditions. In both models, forest soil from oak groves in the municipality of Navacerrada, in the foothills of the Sierra de Guadarrama, Madrid, Spain (40.811938, −3.792028), was used.

The third model corresponded to a field trial carried out at the CEU Montepríncipe experimental site, Boadilla del Monte, Madrid, Spain (40.403826, −3.836913), using one-year-old *Q. ilex* seedlings cultivated until the second year of development was completed.

In the original studies, 35 experimental units per treatment were established. For the present cross-sectional analysis, the data were reorganized according to the plant species, the experimental model, the fertigation matrix, the biological treatment and the type of variable analyzed. The source studies included in the cross-sectional analysis are shown in Table 2.

### 2.4. Considered Treatments in the Source Studies

In the original experimental studies, a factorial design was used based on the combination of a chemical treatment (Table 3), corresponding to the fertigation matrix, and a biological treatment, corresponding to the presence or absence of bacterial inoculation.

For the cross-sectional analysis, comparisons were always made between inoculated treatments and their respective non-inoculated controls within the same fertigation matrix. Therefore, it was avoided to attribute effects to the strain that could depend exclusively on the organic matrix.

### 2.5. Methodological Synthesis of the Source Studies

The integrated data came from trials previously carried out by the research group. This section summarizes the methodologies used to contextualize the origin of the reanalyzed variables.

In the source studies, the organic waste from a wastewater treatment plant (EDAR) was used as a fertigation matrix at a dilution of 1/512 (*v*/*v*), and an autoclaved fraction (EDARST) was also obtained. Bacterial inoculation was performed by adjusting the density of *B. pretiosus* to 0.5 on the McFarland scale (~10^8^ CFU·mL^−1^) and incorporating the suspension into the corresponding fertigation matrix.

At the end of each experiment, biometric variables (weight and shoot length, root length, and number of leaves) were registered and plant material was collected for nutritional analysis. When the available biomass was insufficient for individual determinations, composite samples were used. Nutritional parameters included crude protein, soluble protein, total amino acids, fiber fractions, lignin, starch, soluble sugars, and minerals.

The functional activity of the rhizospheric microbiota was evaluated using Biolog EcoPlates^®^, calculating the Shannon–Weaver diversity index. The phenotypic profile of community antibiotic resistance was determined by a cenoantibiogram, recording MIC values from rhizospheric bacterial suspensions inoculated on Mueller–Hinton agar with antibiotic gradient strips [18]. The bacterial taxonomic composition was characterized by massive amplicon sequencing of the *16S rRNA* gene, obtaining alpha- and beta-diversity metrics [21].

A total of 30 biological samples were included in the microbiome analyses. These samples originated from five independent experiments, with three biological replicates per treatment in each experiment, resulting in 15 samples for the control treatment and 15 samples for the Bacillus pretiosus C1 treatment. These samples were used for alpha-diversity, beta-diversity (PCoA), PERMANOVA, ANOSIM and differential abundance analyses.

### 2.6. Included Variables in the Cross-Sectional Analysis and Harmonization and Organization of the Data

For the cross-sectional analysis, the variables available in the source studies that could be compared from a biological and methodological point of view were selected, grouped into the functional domains of plant biometry, nutritional composition, rhizospheric functional diversity, bacterial taxonomic structure and community profile of antibiotic resistance (Supplementary Table S1). Not all variables were available in all source studies; each domain was analyzed only in the experimental models with equivalent data.

Data were harmonized by a standardized nomenclature for species, matrix type and biological treatment. Experimental groups were coded as combinations of these factors (WC0, WC1, EDARC0, EDARC1, EDARSTC0 and EDARSTC1). All analyses were performed within the same fertigation matrix and, when required, within the same species or experimental model, thereby ensuring the comparability of the datasets and preventing the integration of non-equivalent experimental contexts.

### 2.7. Statistical Analysis and Functional Integration Strategy

Analyses were performed independently for each functional domain, on biologically and methodologically comparable datasets. For biometric and nutritional variables, comparisons were established between inoculated plants (C1) and controls (C0) within each fertigation matrix. When the assumptions of normality and homogeneity of variances (Levene’s test) were met, ANOVA models were applied with calculation of estimated marginal means (*emmeans*) and 95% confidence intervals. When the parametric assumptions were not met, the Kruskal–Wallis test was used with Dunn’s post hoc comparisons and Holm’s correction; the results were expressed as median and interquartile range.

Analyses were performed independently for each functional domain using biologically and methodologically comparable datasets. For biometric and nutritional variables, inoculated plants (C1) were compared with non-inoculated controls (C0) within each fertigation matrix. When assumptions of normality and homogeneity of variances (Levene’s test) were satisfied, data were analyzed using ANOVA models, and estimated marginal means (*emmeans*) with 95% confidence intervals were calculated. For datasets that did not meet parametric assumptions, the Kruskal–Wallis test was applied, followed by Dunn’s post hoc comparisons with Holm correction. The results were presented as the median and interquartile range (IQR).

To explore the multivariate structure of nutritional variables and the antibiotic resistance profile, principal component analysis (PCA) was applied; MIC values were transformed at the log2 scale prior to analysis. Rhizospheric functional diversity was assessed using the Shannon–Weaver index obtained from Biolog EcoPlates^®^ at the point of maximum available metabolic activity. The alpha-diversity of the bacterial microbiota was estimated using Shannon’s index, Pielou’s evenness, Faith’s phylogenetic diversity and number of taxa observed. Beta-diversity was assessed using Weighted UniFrac distances visualized in PCoA, with statistical significance estimated by PERMANOVA (999 permutations), ANOSIM and PERMDISP to distinguish real displacements in community composition from differences due to intragroup dispersion. The differential abundance of taxa was analyzed to identify the bacterial groups that explained the observed differences. All analyses and figures were performed in R v4.5.1 (R Core Team, 2025).

A single mixed-effects model including experiment or study as a random effect was not applied because the source datasets differed in host species, experimental structure, treatment combinations, response variables and analytical platforms. These differences prevented the construction of a single homogeneous model in which observations could be interpreted as repeated measurements of the same experimental design. To avoid treating heterogeneous observations as independent replicates, statistical analyses were performed separately for each comparable dataset or functional block. Principal component analyses were used only as exploratory visualizations, whereas inferential interpretation was based on the corresponding univariate or multivariate statistical tests reported for each block.

## 3. Results

### 3.1. Effect of B. pretiosus on Plant Growth

**Figure 1:**
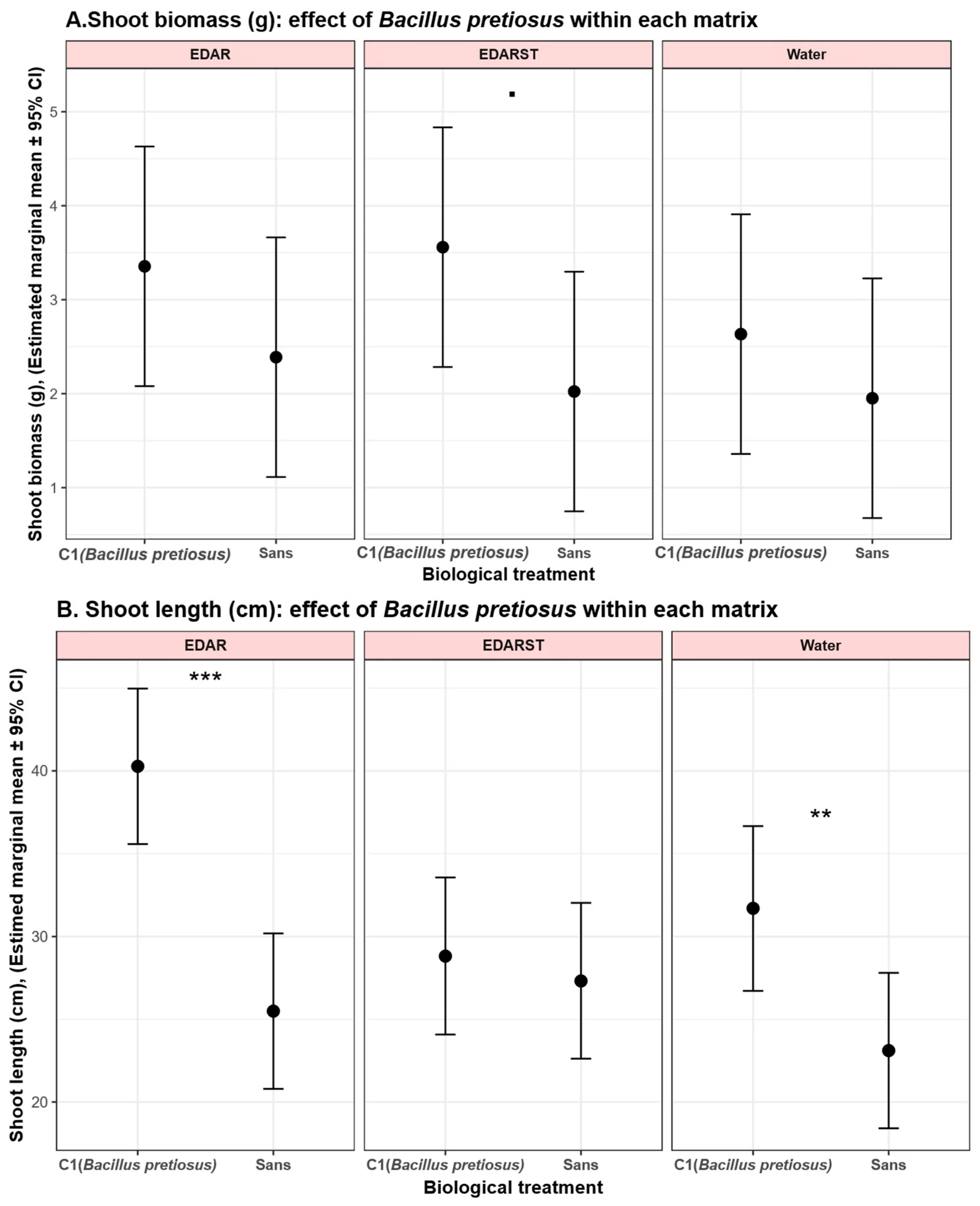
Effect of *Bacillus pretiosus* C1 inoculation on biometric variables of *Quercus* seedlings. (**A**) Shoot biomass (g fresh weight) and (**B**) shoot length (cm). Points represent estimated marginal means (EMM) ± 95% confidence intervals (CIs), derived from ANOVA models with post hoc pairwise comparisons within each fertigation matrix. Significance symbols denote pairwise comparisons between C1 and C0 within each matrix: ** *p* < 0.01; *** *p* < 0.001. W: Water; EDAR, WWTP-derived organic fertigation matrix; EDARST, sterilized WWTP; C0, non-inoculated control; C1, *B. pretiosus* C1-inoculated treatment.

Across all fertigation matrices, inoculation with *Bacillus pretiosus* C1 consistently exerted a positive impact on shoot biomass relative to the non-inoculated controls (C0), although these differences did not reach formal statistical significance (Figure 1A). This shift was most pronounced in the sterilized matrix (EDARST), which exhibited a positive statistical trend near the significance threshold (estimated difference = 1.536 g; *p* = 0.0739). In contrast, under unsterilized organic fertigation (EDAR) and water (Water) conditions, the biofertilizer treatment yielded higher absolute EMM values for biomass—averaging approximately 3.3 g in EDAR and 2.6 g in Water compared to 2.4 g and 1.9 g in their respective controls—yet high within-group variability prevented these increases from achieving statistical significance (*p* > 0.05).

Unlike biomass accumulation, shoot elongation responded much more robustly and consistently to bacterial inoculation (Figure 1B). The PGPR effect was highly matrix-dependent: *B. pretiosus* C1 induced a strong, highly significant increase in shoot length when applied in unsterilized wastewater (EDAR; estimated difference = 14.779 cm; *p* < 0.0001, ***) and in plain water (Water; estimated difference = 8.580 cm; *p* = 0.00583). Interestingly, this growth-promoting effect disappeared in the sterilized matrix (EDARST), where shoot length remained virtually unchanged between inoculated (28.8 cm) and non-inoculated plants (27.2 cm) (*p* > 0.05).

Overall, these findings reveal a clear decoupling between vertical shoot expansion and total biomass accumulation in *Quercus* seedlings during the evaluated stage. While *B. pretiosus* C1 reliably drives apical elongation—particularly when combined with non-sterilized organic effluent (EDAR)—it triggers a milder, non-significant shift toward fresh biomass gain. The stark difference in shoot elongation between EDAR and EDARST further suggests that the growth-promoting activity of *B. pretiosus* C1 may rely on synergy with the native microbiota present in raw effluents, or that heat sterilization alters matrix chemistry in ways that modulate the strain’s phytostimulatory capabilities.

### 3.2. Effect of B. pretiosus on the Plant Nutritional Profile

**Figure 2:**
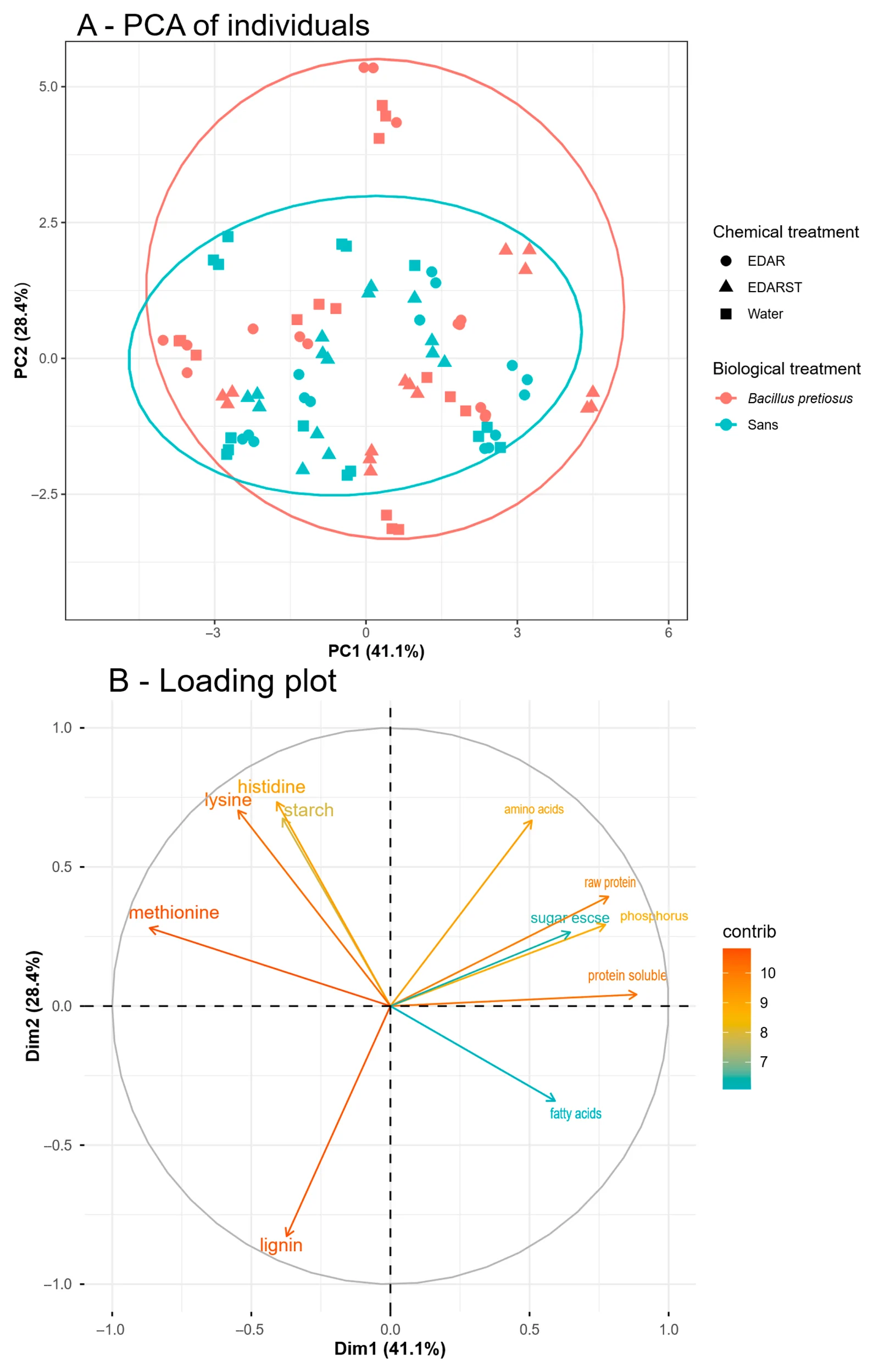
Principal component analysis (PCA) of plant nutritional variables. Samples correspond to *Quercus* seedlings from all fertigation matrices (Water, EDAR, and EDARST) and both biological treatments. (**A**) Score plot: ellipses represent 95% confidence regions for each group. (**B**) Loading plot: variables with the highest absolute loadings along the axes of greatest separation between groups include nitrogen-related nutritional variables (crude protein, soluble protein, and total amino acids). The PCA was performed on standardized (z-scored) nutritional variables. No inferential statistical test was applied to the ordination; the PCA is presented as an exploratory multivariate visualization of the nutritional dataset. C0: non-inoculated control; C1: *B. pretiosus*; EDAR: WWTP-derived organic fertigation matrix; EDARST: sterilized WWTP.

Principal component analysis (PCA; Figure 2) revealed that the first two principal components explained 69.56% of the total variance (PC1 = 41.13%; PC2 = 28.42%). Sample ordination indicated a tendency towards separation between biological treatments, although the overlap observed among groups suggested only a moderate treatment effect.

The PCA suggests that inoculation with *B. pretiosus* is associated with a partial modification of the plant nutritional profile, mainly explained by variables linked to nitrogenous metabolism, such as proteins and amino acids. However, the overlap between groups indicates that the effect is not uniform across all samples and depends on the experimental context or fertigation matrix.

**Figure 3:**
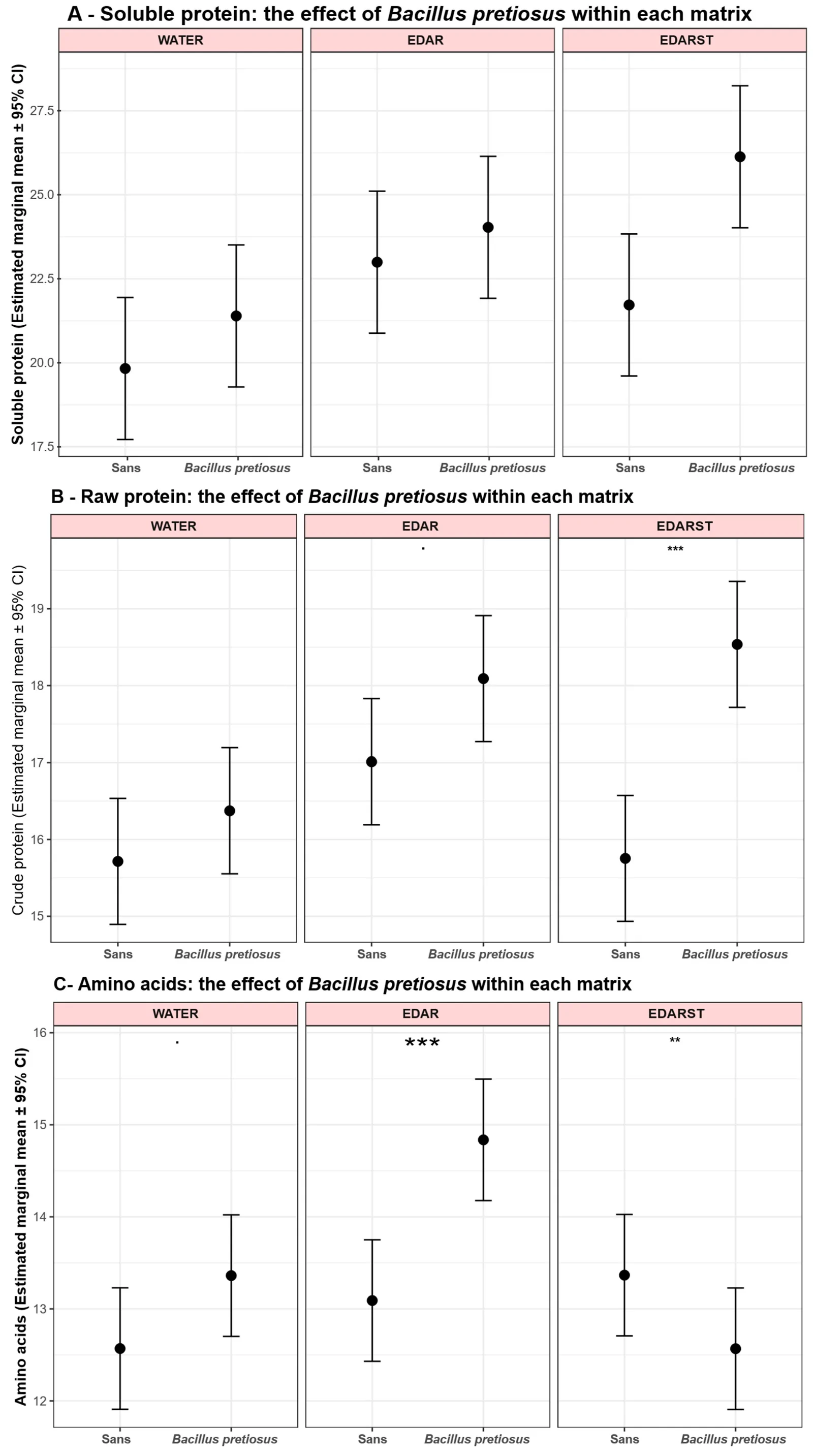
Effect of *Bacillus pretiosus* C1 inoculation on nitrogen-related nutritional variables of *Quercus* seedlings. (**A**) Soluble protein (g per 100 g dry matter), (**B**) crude protein (g per 100 g dry matter), and (**C**) total amino acids (g per 100 g dry matter), as a function of biological treatment and fertigation matrix. Points represent estimated marginal means (EMMs) ± 95% confidence intervals (CIs), derived from ANOVA models with *post hoc* pairwise comparisons between *B. pretiosus*-inoculated plants (C1) and non-inoculated controls (sans) within each fertigation matrix. Significance symbols denote pairwise C1 vs. sans comparisons: ** *p* < 0.01; *** *p* < 0.001. C0: non-inoculated control; C1: *B. pretiosus*; W: Water, EDAR, WWTP-derived organic fertigation matrix; EDARST: sterilized WWTP-derived organic fertigation matrix.

Inoculation with *B. pretiosus* C1 exerted a distinct, matrix-dependent effect on the nitrogenous compounds and nutritional parameters of *Quercus* seedlings (Figure 3).

Regarding soluble protein content (Figure 3A), post hoc analysis revealed that *B. pretiosus* C1 inoculation led to a marked, statistically significant accumulation only when applied within the sterilized matrix (EDARST; estimated difference = 4.597 g/100 g DM; *p* = 0.00238). In contrast, no significant changes were detected in the unsterilized effluent (EDAR; estimated difference = −1.001 g/100 g DM; *p* = 0.4968) or in the water control treatment (WATER; estimated difference = 1.539 g/100 g DM; *p* = 0.2970), despite a subtle positive EMM shift observed in the latter.

A highly consistent response was found for crude protein (Figure 3B). Seedlings inoculated with *B. pretiosus* C1 exhibited a substantial increase in crude protein under EDARST conditions compared to their non-inoculated counterparts (estimated difference = 2.725 g/100 g DM; *p* < 0.0001, ***). A positive, non-significant trend was also detected in the unsterilized EDAR matrix (estimated difference = 0.984 g/100 g DM; *p* = 0.0874), whereas inoculation in plain water yielded no statistically significant difference (*p* = 0.2448).

Finally, the dynamics of total amino acids (Figure 3C) highlighted a contrasting physiological response depending on matrix sterilization. In the unsterilized organic effluent (EDAR), *B. pretiosus* C1 significantly boosted amino acid levels (estimated difference = 1.752 g/100 g DM; *p* = 0.000255, ***), while a marginally significant positive trend was noted in water (estimated difference = 0.799 g/100 g DM; *p* = 0.0848). Remarkably, in the sterilized effluent (EDARST), inoculation induced a significant decline in total amino acid content (estimated difference = −1.434 g/100 g DM; *p* = 0.00242).

Taken together, these results demonstrate that while *B. pretiosus* C1 primarily promotes protein synthesis (both soluble and crude) when combined with a sterilized organic matrix (EDARST), its benefits in raw organic effluent (EDAR) manifest chiefly through the enrichment of the free amino acid pool rather than complex protein accumulation.

Overall, the effects of *B. pretiosus* on plant nutrition were primarily reflected in nitrogen-related parameters. The increases in protein and amino acid contents observed in specific matrices suggest an enhancement of nitrogen acquisition and/or assimilation processes, ultimately contributing to improved nutritional status. These results support the hypothesis that *B. pretiosus* promotes plant nutritional efficiency under the evaluated conditions.

In contrast, variables related to storage carbohydrates and structural components did not show significant changes, suggesting that the nutritional effect of the strain was mainly concentrated in nitrogenous variables.

### 3.3. Effect of B. pretiosus on the Community Profile of Antibiotic Resistance

**Figure 4:**
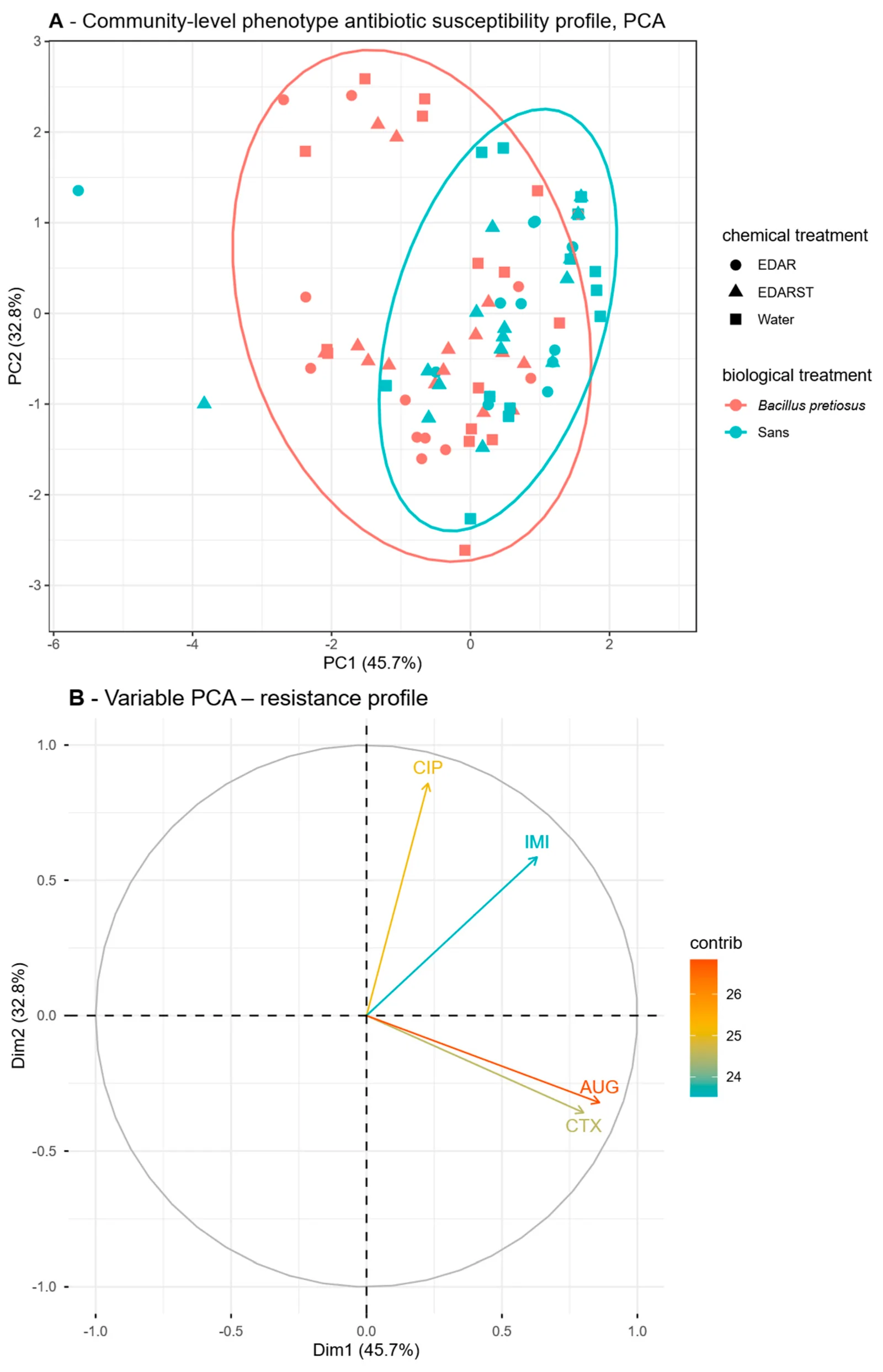
Principal component analysis (PCA) of the community-level phenotypic antibiotic susceptibility profile of the rhizospheric microbiota. MIC values for four antibiotics were log2-transformed prior to PCA. (**A**) Score plot: sample scores along PC1 and PC2, color-coded by biological treatment (non-inoculated control, sans; *B. pretiosus* C1-inoculated) and differentiated by fertigation matrix using point shape or fill as indicated in the legend. (**B**) Loading plot: contribution of the four antibiotic MIC variables (CTX, CIP, IMI, and AUG) to PC1 and PC2, colored according to their squared cosine (cos^2^) contribution to the total variance explained by the two components. The PCA is presented as an exploratory multivariate visualization; no inferential statistical test was applied to the ordination. Abbreviations: PCA, principal component analysis; MIC, minimum inhibitory concentration; CTX, cefotaxime; CIP, ciprofloxacin; IMI, imipenem; AUG, amoxicillin/clavulanic acid; sans, non-inoculated control; *Bacillus pretiosus* C1-inoculated treatment; Water, irrigation control; EDAR, WWTP-derived organic fertigation matrix; EDARST, sterilized WWTP-derived organic fertigation matrix; WWTP, wastewater treatment plant. Small triangles, circles and squares are each replicate. Big triangles, circles and squares are the mean for the replicates.

Principal component analysis (PCA, Figure 4) of the resistance profile included four antibiotics: cefotaxime (CTX), ciprofloxacin (CIP), imipenem (IMI), and amoxicillin/clavulanic acid (AUG). The first two components explained 78% of the total variation in the transformed MIC profile at the log_2_ scale. The graph of variables showed that CTX, IMI and AUG contributed significantly to the segregation of the samples, while CIP showed a differential contribution compared to the rest of the antibiotics.

Principal component analysis indicated a partial structuring of community-level antibiotic susceptibility profiles according to biological treatment. The observed separation was primarily driven by cefotaxime, IMI and AUG, which exhibited the highest loadings on the principal components. These antibiotics were therefore selected for subsequent inferential analyses.

**Figure 5:**
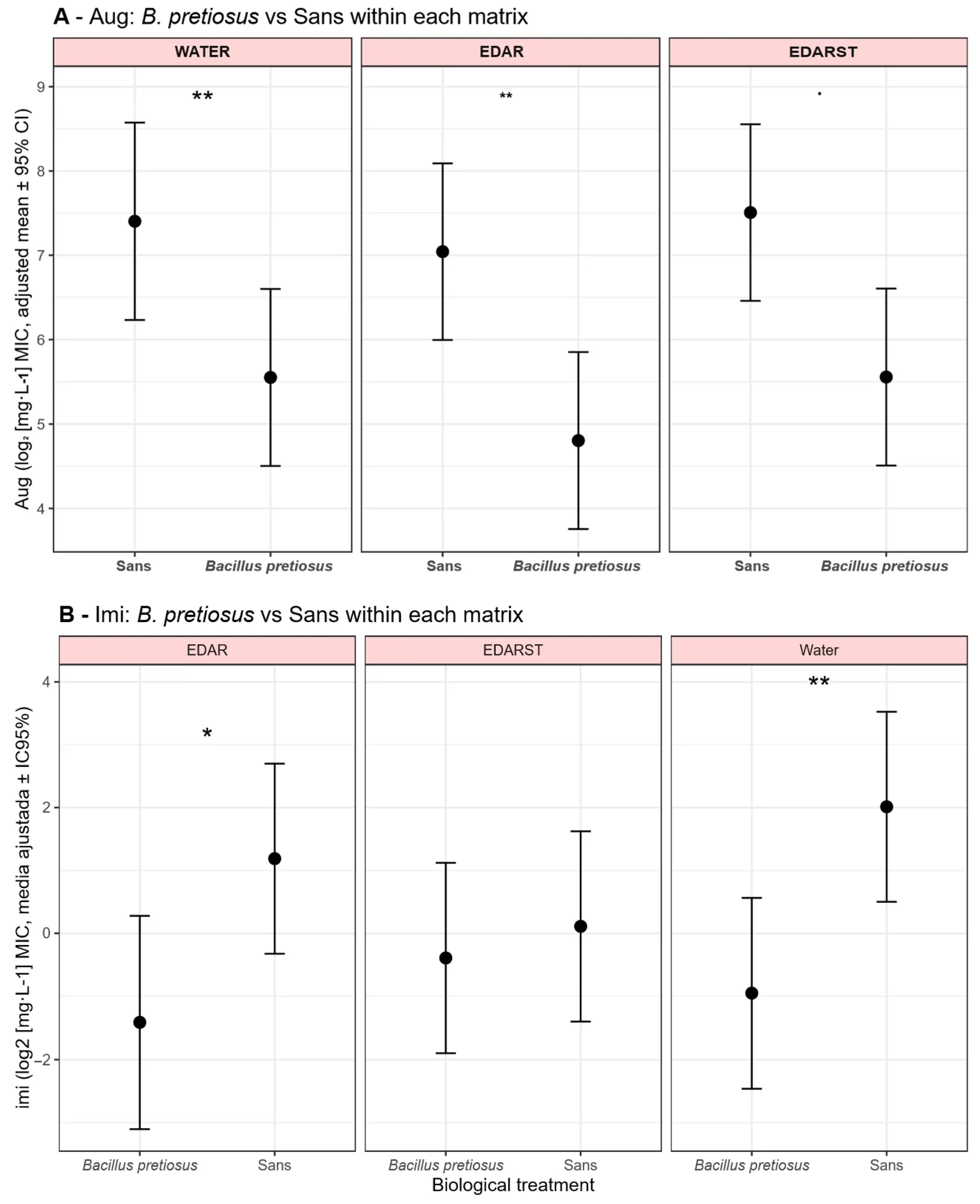
Post hoc pairwise comparisons of log2-transformed minimum inhibitory concentration (MIC) values for (**A**) amoxicillin/clavulanic acid (AUG) and (**B**) imipenem (IMI), between *B. pretiosus* C1-inoculated plants and non-inoculated controls (sans) within each fertigation matrix. Points represent estimated marginal means (EMMs) ± 95% confidence intervals (CIs) of log2 (MIC) values, derived from ANOVA models with post hoc pairwise comparisons within each matrix. Significance symbols denote pairwise C1 vs. C0 comparisons within each matrix: * *p* < 0.05; ** *p* < 0.01. Note that lower log2 (MIC) values correspond to lower MICs and therefore to greater antibiotic susceptibility in the rhizospheric community. Abbreviations: MIC, minimum inhibitory concentration (mg·L^−1^); AUG, amoxicillin/clavulanic acid; IMI, imipenem; EMM, estimated marginal mean; CI, confidence interval; C0, non-inoculated control; C1, *B. pretiosus* C1-inoculated treatment; Water, irrigation control; EDAR, WWTP-derived organic fertigation matrix; EDARST, sterilized WWTP-derived organic fertigation matrix; WWTP, wastewater treatment plant.

Inoculation with *Bacillus pretiosus* C1 significantly altered the antibiotic susceptibility profile of the rhizospheric microbial community, although this response was highly antibiotic- and matrix-dependent (Figure 5). For cefotaxime (CTX), a general non-significant trend toward lower MIC values was observed in inoculated samples (*p* = 0.0812), with no individual matrix reaching statistical significance. Similarly, ciprofloxacin (CIP) levels remained unaffected by either the biological treatment or its interaction with the fertigation matrix.

In contrast, amoxicillin/clavulanic acid (AUG) susceptibility was strongly modulated by the biological treatment (*p* = 0.000118), with *B. pretiosus* C1 inoculation consistently shifting the rhizospheric community toward lower log_2_ (MIC) values (Figure 5A). Post hoc pairwise comparisons revealed significant MIC reductions in both unsterilized organic effluent (EDAR; estimated difference = −2.163; *p* = 0.00761) and plain water (Water; estimated difference = −1.974; *p* = 0.00967). In the sterilized matrix (EDARST), inoculation induced a similar downward trend in EMM values, though it reached only marginal statistical significance (estimated difference = −1.267; *p* = 0.0928).

A comparable matrix-dependent effect was recorded for imipenem (IMI; main treatment effect *p* = 0.00232), where lower $\log_{2}\left(\mathrm{MIC}\right)$ values indicated heightened susceptibility in inoculated rhizospheres (Figure 5B). Pairwise tests confirmed that *B. pretiosus* C1 significantly decreased IMI MICs under unsterilized organic fertigation (EDAR; estimated difference = −2.600; *p* = 0.0251, *) and water irrigation (Water; estimated difference = −2.962; *p* = 0.00722). Conversely, no significant differences were detected in the sterilized matrix (EDARST; *p* > 0.05), where EMM values remained low and virtually identical between control and inoculated treatments.

Overall, these results demonstrate that *B. pretiosus* C1 inoculation selectively enhances rhizospheric susceptibility (reducing MICs) to β-lactams (AUG and IMI), particularly when applied in non-sterilized organic effluent (EDAR) or water. The dampening of this response in EDARST highlights the key role of native effluent microbiota and matrix sterilization in mediating biofertilization-induced shifts in resistome dynamics.

The results indicate that inoculation with *B. pretiosus* was not associated with an increase in community-level phenotypic antibiotic resistance. In contrast, significant reductions in MIC values for IMI and AUG were observed in specific matrices, suggesting a shift towards greater antibiotic susceptibility within the rhizospheric bacterial community. Collectively, these findings are consistent with a favorable biosafety profile of *B. pretiosus* in terms of its impact on community antimicrobial resistance.

### 3.4. Effect of B. pretiosus on Rhizospheric Functional Diversity

The Shannon–Weaver diversity index remained high across all treatments at 144 h, as evaluated by Biolog EcoPlate^®^ community-level physiological profiling. Overall, treatments yielded a mean value of $H^{\prime }=4.59\pm 0.23$, with individual values corresponding to 4.71±0.13 for WC0, 4.48±0.25 for WC1, 4.37±0.0 for EDARC0, 4.75±0.03 for EDARC1, 4.72±0.14 for EDARSTC0, and 4.44±0.22 for EDARSTC1 (where values represent mean $H^{\prime }\pm$ standard deviation). These values ranged from $H^{\prime }=4.35$ to $H^{\prime }=4.79$ across treatments, reflecting a high degree of functional diversity and metabolic evenness within the rhizospheric bacterial communities. No statistically significant differences were detected between chemical treatments (water, EDAR or EDARST) or between biological treatments (control without inoculation and *B. pretiosus*). Therefore, the incorporation of the strain (C1), regardless of the chemical matrix used, did not significantly modify the overall functional diversity of the rhizospheric communities. Overall, inoculation with *B. pretiosus* was not associated with detectable changes in the functional diversity of the rhizospheric bacterial community, which consistently exhibited high levels of metabolic diversity after 144 h of incubation.

### 3.5. B. pretiosus Effect on the Taxonomic Structure of the Rhizospheric Microbiota

The Pielou Evenness Index, used as an alpha-diversity metric, showed no significant differences associated with inoculation with *B. pretiosus*, chemical treatment, or the interaction between both factors. These results indicate that the incorporation of C1 did not significantly alter the uniformity of the bacterial community, maintaining a balanced distribution of alpha-diversity between the different treatments.

**Figure 6:**
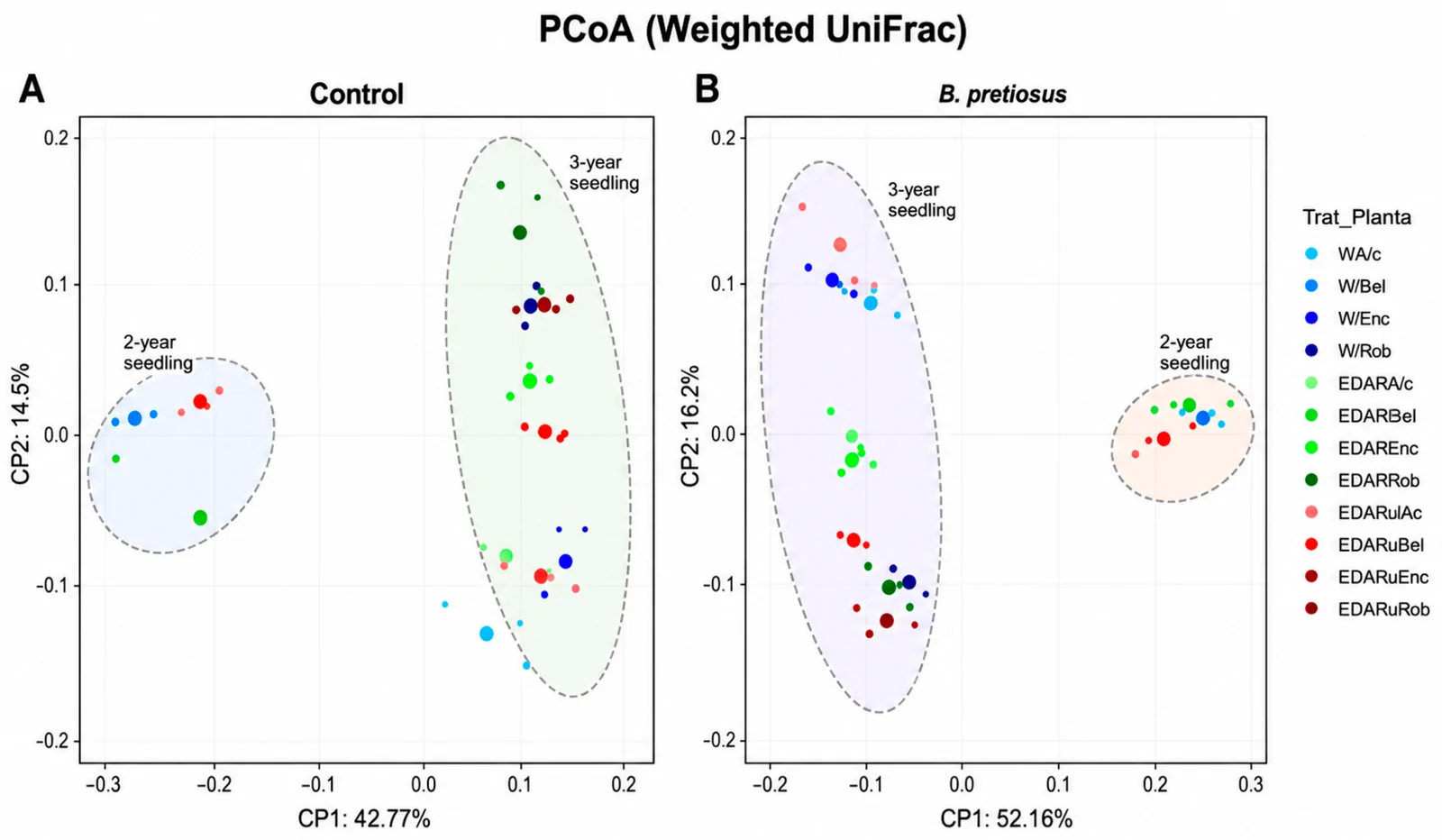
Principal coordinates analysis (PCoA) of rhizospheric bacterial community composition, based on Weighted UniFrac distances calculated from 16S rRNA gene amplicon sequencing data. (**A**) Non-inoculated control plants (C0). (**B**) *B. pretiosus* C1-inoculated plants (C1). In both panels, points represent individual samples color-coded by plant age (2-year-old and 3-year-old seedlings). Ellipses indicate 95% confidence regions for each age group. Percentages on each axis indicate the proportion of total variance explained by that coordinate. The ordination pattern should be interpreted as exploratory and hypothesis-generating rather than as confirmatory evidence of community-level differentiation. Abbreviations: PCoA, principal coordinates analysis; CP1/CP2, first and second principal coordinates; Weighted UniFrac, abundance-weighted phylogenetic distance metric; C0, non-inoculated control; C1, *B. pretiosus* C1-inoculated treatment. Small circles are each replicate. Big circles are the mean for the replicates.

In order to evaluate possible changes in the composition of the bacterial communities, beta-diversity was analyzed by PCoA based on Weighted UniFrac, PERMANOVA, ANOSIM and PERMDISP distances. Principal coordinates analysis (PCoA; Figure 6) revealed a clear influence of sapling age on community structure, with distinct clustering patterns between control and *B. pretiosus*-inoculated plants. Samples from 2- and 3-year-old saplings formed separate groups in both treatments. However, the ordination pattern differed following inoculation, suggesting an effect of *B. pretiosus* on the spatial organization of the microbial community.

Statistical analyses showed no significant differences between the groups defined by the treatment. The PERMANOVA analysis detected no significant effects (F = 7.30; R^2^ = 0.777; *p* = 1), even though the model explained a high proportion of the total variation. Consistently, the ANOSIM analysis showed a high apparent separation between groups (R = 0.999), but without statistical significance (*p* = 1).

Likewise, the dispersion analysis (PERMDISP) did not show significant differences between groups (F = 1.378; *p* = 0.248), indicating that intragroup variability was comparable between treatments. Taken together, these results suggest that the incorporation of biological treatment did not produce significant changes in the overall structure of the microbial community.

Although PCoA ordering showed partial visual separation between groups, the PERMANOVA and ANOSIM analyses did not confirm statistically significant differences in the overall community structure. Therefore, the results should be interpreted as a possible trend in community reorganization, not as a statistically demonstrated modification of beta-diversity.

**Figure 7:**
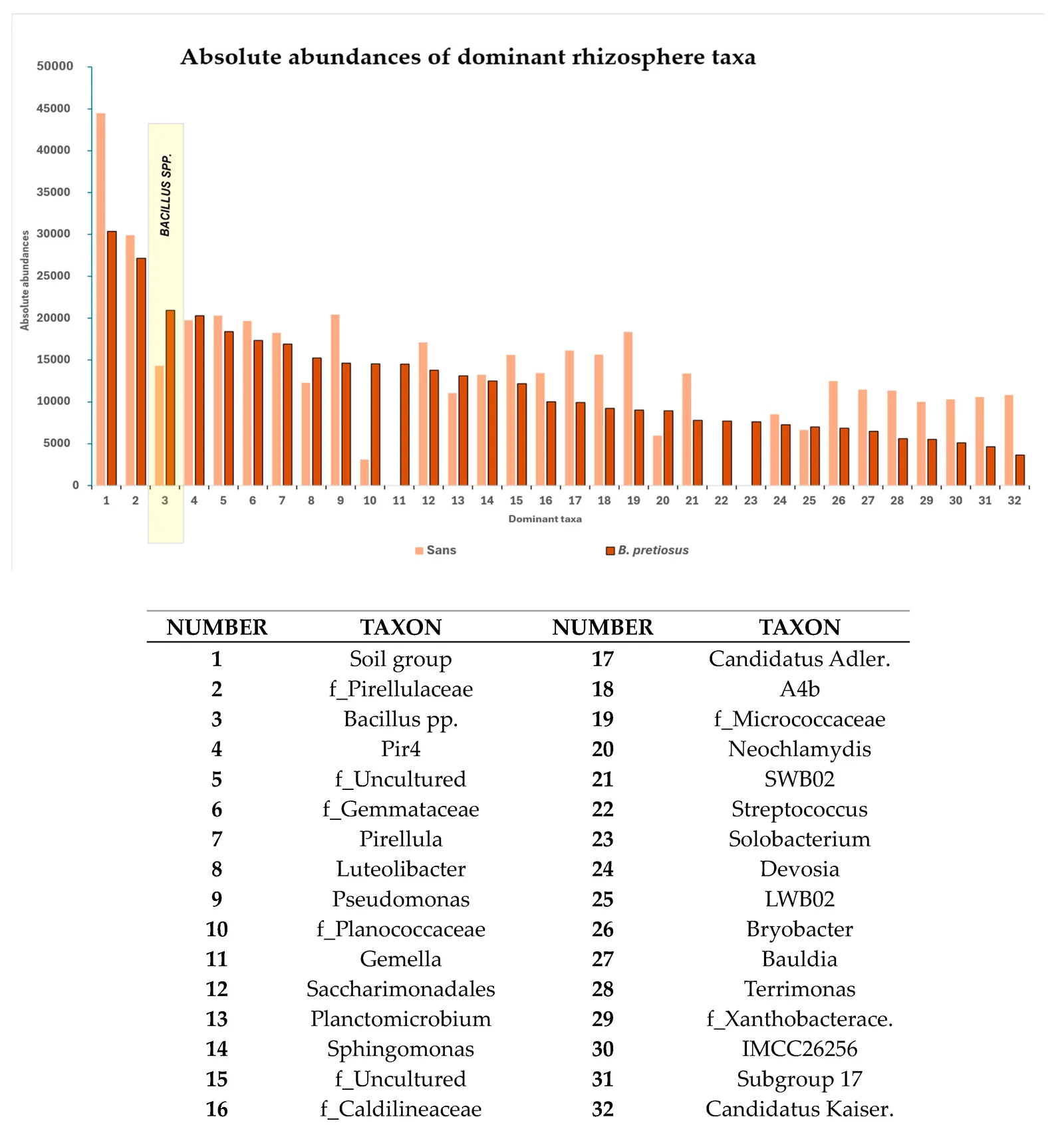
Taxonomic abundances of the dominant rhizospheric bacterial taxa in non-inoculated control plants (C0) and *B. pretiosus* C1-inoculated plants (C1), based on 16S rRNA gene amplicon sequencing of rhizospheric DNA. Bars represent the mean read abundance (or relative abundance, as appropriate to the normalization applied; see Methods) of each taxon per treatment. Taxa shown were selected from those with the highest mean abundance in at least one of the two treatment groups. Each *x*-axis numeric code corresponds to a specific taxon as listed in the legend table embedded in the figure. The bar corresponding to Bacillus spp. is highlighted in yellow for visual reference. Abbreviations: Sans (C0), non-inoculated control; C1, *B. pretiosus* C1-inoculated treatment.

To identify the specific microbial drivers underlying the structural separation observed in the β-diversity analysis, differential abundance was evaluated across the dominant rhizospheric bacterial taxa (Figure 7). Taxonomic profiling revealed distinct shifts in the absolute abundance of several key operational taxonomic units (OTUs) between non-inoculated control plants (*Sans*) and *B. pretiosus* C1-inoculated plants.

As anticipated, the most prominent shift linked to the biofertilizer treatment occurred within taxon 3 (*Bacillus* spp.), which experienced a substantial relative enrichment in C1-inoculated rhizospheres (Figure 7, highlighted in yellow). While control plants maintained a baseline abundance of approximately 14,300 reads for *Bacillus* spp., inoculated plants displayed a marked increase, reaching over 20,900 reads. This ~46% expansion confirms the effective colonization and persistent rhizosphere establishment of the inoculated strain.

Beyond *Bacillus* spp., the biological treatment noticeably restructured the broader bacterial community hierarchy. In control rhizospheres (*Sans*), the dominant community was heavily driven by high-abundance taxa such as *Soil group* (1; ~44,500 reads), *f_Pirellulaceae* (2; ~29,800 reads), *Pseudomonas* (9; ~20,400 reads), *f_Micrococcaceae* (#19; ~18,300 reads), and *Bryobacter* (#26; ~12,400 reads). Following *B. pretiosus* C1 inoculation, many of these background taxa exhibited moderate reductions in abundance—most notably *Soil group* (1; declining to ~30,300 reads), *Pseudomonas* (9; dropping to ~14,600 reads), and several low-abundance groups including *Bryobacter* (26) and *f_Xanthobacteraceae* (29).

Conversely, *B. pretiosus* C1 inoculation selectively promoted specific bacterial groups, such as *f_Planococcaceae* (10), which surged from negligible baseline levels (~3000 reads in *Sans*) to over 14,500 reads in treated plants, as well as *Luteolibacter* (8; increasing from ~12,200 to ~15,200 reads).

Overall, these taxonomic patterns demonstrate that *B. pretiosus* C1 inoculation does not merely introduce a transient strain; rather, it triggers a broader ecological reorganization of the *Quercus* rhizosphere, successfully establishing *Bacillus* spp. while reshaping the abundance of coexisting dominant taxa.

This pattern is consistent with the previously obtained alpha- and beta-diversity results, indicating that the incorporation of *B. pretiosus* acts mainly through a point modulation of certain microbial groups rather than through a massive restructuring of the rhizospheric microbiome.

### 3.6. Cross-Sectional Synthesis of the Functional Profile of B. pretiosus

Taken together, the results obtained for plant growth, nutritional status, antibiotic resistance and rhizospheric microbial communities provide an integrated view of the overall effects of *B. pretiosus* inoculation (Supplementary Table S2). A comprehensive summary of the statistical analyses, effect estimates, and corresponding *p*-values for all response variables is provided in Supplementary Table S3.

Overall, the evidence collected shows that inoculation with *B. pretiosus* was associated with positive effects on plant growth and plant nutritional profile, while functional diversity, microbial diversity, and overall rhizospheric community structure remained stable. Moreover, a higher relative abundance of *Bacillus* spp. was observed in the inoculated treatments and no increase in the community profile of antibiotic resistance was detected. The integration of these results defines a functional profile characterized by plant growth promotion and rhizospheric persistence, accompanied by a slight alteration of the evaluated microbiological properties.

## 4. Discussion

### 4.1. Integrated Interpretation of the Functional Profile of Bacillus pretiosus C1

The cross-sectional synthesis presented in this study integrates evidence from multiple independent experimental datasets to provide an initial assessment of the functional and ecological profile of *Bacillus pretiosus* C1 as a plant growth-promoting bacterium (PGPB) in species of the genus *Quercus*. Across the functional domains evaluated, such as plant growth, nutritional composition, rhizospheric functional diversity, bacterial community structure, and community-level phenotypic antibiotic susceptibility, the results collectively suggest a promising but context-dependent functional profile.

The most consistent plant-associated responses were related to shoot elongation and improvements in nitrogen-related nutritional variables, particularly under WWTP-derived fertigation matrices (EDAR and EDARST). In contrast, inoculation was not associated with statistically supported alterations in rhizospheric functional diversity, global bacterial community structure, or the evaluated community-level phenotypic antibiotic resistance profile. Taken together, these findings suggest that *B. pretiosus* C1 can promote plant performance while maintaining key microbiological attributes commonly associated with soil health-oriented management strategies.

However, the interpretation of these findings must remain cautious. The present work is not a prospective controlled trial or a formal meta-analysis, but a harmonized cross-sectional synthesis of previous experimental datasets generated under heterogeneous conditions. Therefore, the results should be interpreted as convergent evidence supporting the functional potential of *B. pretiosus* C1, rather than as definitive proof of efficacy or mechanism. This framework provides a basis for hypothesis generation and for the design of future prospective validation trials.

### 4.2. Effects on Plant Growth and Nutritional Composition

Among the biometric variables evaluated, shoot elongation was the most consistently supported response associated with inoculation with *B. pretiosus* C1. Significant increases in shoot length were observed in the Water and EDAR fertigation matrices, whereas the response in EDARST was less pronounced. By contrast, shoot biomass showed only non-significant positive trends across most experimental contexts. This asymmetry between shoot length and biomass accumulation is biologically plausible in studies involving woody or long-cycle plant species. Early elongation responses may become detectable before significant changes in total biomass are observed, particularly when experiments are conducted over relatively limited developmental windows. In this context, shoot length appears to be the most sensitive biometric indicator of the plant growth-promoting response associated with *B. pretiosus* C1 under the conditions evaluated. Comparable responses have been reported for other PGPB inoculants, where the magnitude and consistency of plant growth promotion depend strongly on the physicochemical and biological context of the soil or substrate [26,27].

At the nutritional level, the response associated with *B. pretiosus* C1 was mainly concentrated in nitrogen-related variables. Significant increases in crude protein, soluble protein, and total amino acid content were detected in specific fertigation matrices, principally EDAR and EDARST. In contrast, variables related to storage carbohydrates and structural components did not show consistent significant changes. This selective pattern suggests that inoculation may be associated with changes in nitrogen assimilation, protein synthesis, or amino acid accumulation in plant tissues. These findings are consistent with the described effects of other PGPBs of the genus *Bacillus* [12,13]. Nevertheless, the present design does not allow the underlying mechanisms to be established.

The functional traits previously described for *B. pretiosus* C1, including indole-3-acetic acid (IAA) production and siderophore synthesis, provide a plausible biological background for its selection as a PGPB candidate. These traits have been associated in the broader PGPB literature with improved plant growth and nutrient acquisition [15,28,29]. However, in the present study, they should be interpreted only as previously characterized in vitro traits, not as mechanisms directly demonstrated under the experimental conditions integrated here. Their actual contribution to the nutritional changes observed in *Quercus* plants would require targeted prospective assays.

Importantly, the nutritional effects were not homogeneous across all fertigation conditions. The strongest responses were observed in WWTP-derived matrices, whereas the Water matrix showed fewer significant nutritional changes. This indicates that the functional expression of *B. pretiosus* C1 is strongly modulated by the application matrix. Previous studies from the same research line using *B. pretiosus* C1 in other plant systems and WWTP-derived matrices have also reported changes in plant nutritional composition associated with inoculation [29,30,31,32]. Although differences in host species, developmental stage, and experimental context limit direct extrapolation, these findings support the biological plausibility of a nitrogen-associated functional response under organic fertigation regimes.

### 4.3. Role of the Fertigation Matrix in Modulating the Response

One of the central findings of this synthesis is that the response associated with *B. pretiosus* C1 was strongly modulated by the fertigation matrix. The three matrices evaluated represented distinct biological and physicochemical contexts, ranging from a minimal water-based system to WWTP-derived organic fractions with or without a viable resident microbiota. These differences suggest that the effects of the inoculant are context-dependent outcomes resulting from interactions among the bacterium, the plant, the resident microbiota, and the surrounding rhizospheric environment.

The differential responses observed between EDAR and EDARST suggest that the functional outcome of *B. pretiosus* C1 inoculation depends not only on nutrient availability but also on the biological context provided by the fertigation matrix. The contrasting responses between matrices containing or lacking a viable resident microbiota are consistent with the view that PGPB performance is shaped by interactions among the inoculant, the native microbial community, and the physicochemical characteristics of the application environment [29,32,33]. These findings reinforce the need to evaluate microbial inoculants together with their delivery matrix rather than as isolated biological agents. In the context of WWTP-derived residues, the final biofertilizer effect is likely to emerge from interactions among the organic matrix, the introduced PGPB, and the resident microbiota, highlighting the importance of considering both nutrient and microbial components when developing sustainable biofertilization strategies.

### 4.4. Rhizospheric Functional Diversity and Bacterial Community Structure

Inoculation with *B. pretiosus* C1 was not associated with detectable changes in the functional metabolic diversity of the rhizospheric microbiota, as assessed by Biolog EcoPlate™ assays. Shannon–Weaver H′ values remained consistently high across treatment combinations, indicating maintenance of a broad substrate utilization capacity within the bacterial community. From an ecological perspective, this finding suggests that the introduction of *B. pretiosus* C1 did not produce a detectable disturbance in the community-level functional profile evaluated, despite the plant-associated benefits observed in growth and nutritional variables. Such a pattern is particularly relevant for microbial inoculants intended for forest restoration, where enhancement of plant performance should ideally occur without compromising key ecological attributes of the native rhizosphere microbiome. Similar responses have been reported for other PGPB inoculants, in which plant-associated benefits or localized taxonomic shifts occurred without substantial alterations in global functional diversity indicators [32,34,35]. This apparent maintenance of community-level functionality may reflect a degree of ecological resilience within the rhizosphere microbiome, although this interpretation should remain restricted to the endpoint assessed here, as Biolog EcoPlate™ assays provide only a partial representation of the functional complexity of soil microbial communities.

At the taxonomic level, inoculation with *B. pretiosus* C1 was not associated with significant changes in alpha- or beta-diversity indicators. The Pielou Evenness Index suggested that bacterial taxa remained relatively evenly distributed across treatments, while the partial visual organization observed in the PCoA based on Weighted UniFrac distances was not statistically supported by PERMANOVA or ANOSIM. Likewise, PERMDISP analysis indicated that the observed ordination patterns were not primarily driven by differences in within-group dispersion. Taken all together, these results suggest that *B. pretiosus* C1 inoculation did not cause detectable disruption of the global rhizospheric bacterial community structure under the analytical framework applied.

Importantly, this apparent taxonomic stability occurred alongside improvements in plant growth and nitrogen-related nutritional variables, indicating that plant-associated benefits may be achieved without compromising key ecological attributes of the rhizosphere microbiome. Such a pattern is particularly relevant for restoration-oriented biofertilization strategies and may reflect a degree of ecological resilience within the resident microbial community, whereby plant performance can be enhanced without substantial alteration of community structure. Similar resilience patterns have been reported in soil microbial communities exposed to repeated inoculation events, where transient community shifts were followed by recovery towards a stable resident microbiome [36]. Moreover, the maintenance of rhizosphere microbial stability is increasingly recognized as a key component of plant–microbe interactions supporting nutrient cycling and ecosystem functioning in forest soils [36,37].

Differential abundance analysis revealed enrichment of *Bacillus* spp. in inoculated treatments relative to non-inoculated controls. Although the resolution of 16S rRNA gene amplicon sequencing precludes strain-level confirmation of the persistence or activity of *B. pretiosus* C1, this pattern is consistent with a detectable rhizosphere response to inoculation. Similar genus-level enrichment has been reported for other *Bacillus*-based biofertilizers and PGPB inoculants, where selective taxonomic responses occurred without major alterations in overall bacterial diversity or community structure [21,37]. Accordingly, a more appropriate interpretation is that inoculation generated a localized genus-level signal rather than a detectable disruption of the global rhizospheric bacterial community. This coexistence of selective taxonomic enrichment and overall community stability is generally considered a desirable ecological outcome, given the importance of microbial diversity for sustaining soil ecosystem functioning and resilience [22].

### 4.5. Community-Level Phenotypic Antibiotic Susceptibility Profile and Biosafety: One Health Approach

The ecological safety of microbial inoculants is an increasingly important consideration in restoration-oriented biofertilization strategies, where improvements in plant performance should ideally occur without generating undesirable effects on the resident microbiota or associated ecosystem functions. Within this context, the One Health framework highlights the close interconnection between environmental, animal, and human health, emphasizing the need to incorporate microbiological biosafety criteria into the evaluation of new PGPBs [18,34]. The assessment of the community-level phenotypic antibiotic susceptibility profile showed that inoculation with *B. pretiosus* C1 was not associated with an increase in the resistance profile of the culturable rhizospheric fraction. This was evaluated through minimum inhibitory concentration (MIC) values for cefotaxime (CTX), ciprofloxacin (CIP), imipenem (IMI), and amoxicillin/clavulanic acid (AUG). In the current context of increasing concern about the dissemination of antimicrobial resistance through agricultural inputs, organic amendments, and microbial inoculants, this finding represents a relevant biosafety observation.

Organic fertilizers and WWTP-derived amendments have been associated in some contexts with the dissemination of antibiotic resistance genes or resistant bacterial populations in soil ecosystems [38]. Against this background, the absence of increased phenotypic resistance following inoculation with *B. pretiosus* C1 is a favorable indicator. This suggests that, under the evaluated conditions and for the tested antibiotics, inoculation was not associated with an increase in the community-level phenotypic resistance profile. Moreover, the significant reductions in MIC values observed for imipenem (IMI) and amoxicillin/clavulanic acid (AUG) in specific fertigation matrices further support the absence of a generalized resistance-promoting effect. However, the mechanisms underlying these changes cannot be determined from the present study design. They may reflect indirect changes in the culturable community, shifts in the relative abundance of taxa with different intrinsic susceptibility profiles, or interactions with the fertigation matrix. These possibilities remain speculative and require dedicated prospective investigation.

The biosafety assessment of PGPB candidates should be aligned with a One Health perspective, particularly when microbial inoculants are intended for application in environmental, agricultural, or forest restoration settings. In this context, the absence of increased community-level phenotypic resistance provides a useful first-order biosafety indicator for *B. pretiosus* C1, compatible with its potential application as a plant growth-promoting bacterium in forest restoration and biofertilization programs [39]. Nevertheless, the approach used here evaluates only the culturable fraction of the rhizospheric community and only phenotypic susceptibility to the antibiotic panel included in the assay.

### 4.6. Methodological Considerations and Limitations of Cross-Sectional Analysis

Several limitations should be considered when interpreting the results of this synthesis. The underlying datasets originated from independent experiments that differed in plant material, fertigation matrices, experimental conditions, and measured variables. Although these studies addressed a common research question, such methodological heterogeneity may have contributed to variability in the observed responses. Accordingly, the conclusions are best interpreted as consistent trends across multiple experimental contexts rather than definitive effects under a single set of conditions.

A second limitation is the non-uniform availability of variables across source studies. Biometric and nutritional variables were more widely represented, whereas microbiological endpoints, including 16S rRNA gene amplicon sequencing, were available only for specific experimental models. This unequal availability of data limits the degree to which all functional domains can be fully integrated and compared across the entire dataset. The interpretation of microbiome data is also constrained by the resolution of the methodological approach. 16S rRNA gene amplicon sequencing provides useful information on bacterial community structure but does not allow reliable strain-level tracking. Therefore, the observed enrichment of *Bacillus* spp. in inoculated treatments cannot be used to confirm persistence of *B. pretiosus* C1 but can only be used as a guideline. Future studies should incorporate strain-specific molecular markers, cultivation-based recovery, or genomic approaches capable of resolving the inoculant at the strain level.

Despite these limitations, the results consistently associate *B. pretiosus* inoculation with specific plant and microbial responses. However, they do not allow causal relationships or the underlying biological mechanisms to be established, which will require validation through dedicated prospective studies.

### 4.7. Biotechnological Implications and Future Perspectives

The functional profile described in this synthesis supports the continued evaluation of *B. pretiosus* C1 as a candidate PGPB for biofertilization strategies aimed at supporting the establishment and early development of *Quercus* species in forest restoration programs. The most consistent responses (shoot elongation and improvements in nitrogen-related nutritional variables under WWTP-derived fertigation matrices) are relevant for the early stages of seedling production, where initial growth and nutritional status may influence later establishment capacity.

The selection of *B. pretiosus* C1 as a biotechnological candidate is supported by a multi-level evidence base that includes previously described PGPB traits, such as IAA production and siderophore synthesis, together with a genomic biosafety profile characterized by the absence of transmissible antibiotic resistance genes and relevant virulence factors. The present synthesis adds an applied ecological layer to this evidence by showing plant-associated responses without detectable disruption of the evaluated rhizospheric functional diversity indicators or increase in the community-level phenotypic resistance profile. This combination of functional potential and preliminary biosafety evidence is consistent with the criteria proposed for the selection of microbial inoculants intended for environmental applications [40,41,42].

The use of WWTP-derived matrices as fertigation vehicles for *B. pretiosus* C1 also aligns with circular economy approaches based on the agronomic valorization of organic residues. The differential responses observed between EDAR and EDARST indicate that both the chemical composition and the viable microbial component of the matrix can influence the final biological outcome. This has practical implications for the development of biofertilizer products, since the performance of the inoculant may depend on the characteristics of the carrier or fertigation matrix used for its application.

The results of this study should be considered hypothesis-generating and should guide the design of future prospective validation studies. Such studies should include replicated greenhouse and field trials, adequate sample sizes for microbiome analyses, strain-specific tracking of *B. pretiosus* C1, and direct measurement of plant physiological and nutritional responses. They should also incorporate genotypic assessment of antimicrobial resistance genes and mobile genetic elements to complement the phenotypic susceptibility data presented here. Evaluating the consistency of the observed effects across *Quercus* species, developmental stages, soil types, environmental conditions, and application regimes will be essential for determining the ecological robustness and operational transferability of *B. pretiosus* C1 as a biofertilization tool for Mediterranean forest restoration.

## 5. Conclusions

The integration and harmonization of previous experimental data allowed us to transversely evaluate the functional profile of *B. pretiosus* in the genus *Quercus*, showing that its inoculation is associated with a positive biometric response—mainly reflected in the increase in bud length, although with an effect on biomass dependent on the fertigation matrix—and with a clear nutritional impact on nitrogen metabolism (crude protein, soluble protein and total amino acids), though by a physiological mechanism not yet determined. Also, inoculation did not significantly alter the diversity or taxonomic structure of the rhizosphere microbiota—maintaining a detectable presence of *Bacillus* spp. compatible with the inoculum and its potential persistence—nor did it increase community antibiotic resistance according to the cenoantibiogram; even reductions in MIC were observed for certain molecules, all of which consolidate *B. pretiosus* as a PGPB of high interest for biofertilization and forest restoration by combining favorable effects on the plant with strong ecological and microbiological compatibility.

## Figures and Tables

**Table 1:** Identification, functional characteristics, and biosafety profile of *Bacillus pretiosus* C1, subject to cross-sectional analysis.

Category	Information
Strain	*Bacillus pretiosus* C1
Deposit in collections	CECT30674T/DSM 114702T
GenBank code	JAOXJG000000000
Origin group	MICROAMB Collection, CEU San Pablo University
In vitro PGPB characteristics	Production of indoleacetic acid and siderophores
IAA production	Value 5.61 ± 0.03 μg·mL^−1^
Siderophore production	Positive
ACC deaminase activity	Not determined
Genomic biosecurity assessment	Absence of transmissible antibiotic resistance genes and relevant virulence factors
Biotechnological interest	Candidate strain for biofertilization and ecological restoration

**Table 2:** Source experimental studies included in the cross-sectional harmonized synthesis.

Source Studies	Species/Model	Experimental Model and Setting	Extracted Variables
Fernández-Pastrana et al. 2025 [24]	*Quercus ilex*	Field/natural conditions	Biometrics, nutritional, 16S rRNA seq.; cenoantibiogram; functional diversity.
Fernández-Pastrana et al. 2025 [17]	*Quercus ilex*	Controlled laboratory conditions	
González-Reguero et al. 2026 [25]	*Quercus suber*		
Penalba-Iglesias et al. 2025 [16]	*Quercus pyrenaica*		

Abbreviations: 16S rRNA seq., 16S rRNA gene amplicon sequencing; cenoantibiogram, community-level phenotypic antibiotic susceptibility profiling based on minimum inhibitory concentration (MIC) determination. Functional diversity was assessed using Biolog EcoPlate^®^ (Hayward, CA, USA) community-level physiological profiling.

**Table 3:** Chemical (fertigation matrix) and biological (inoculation) treatments applied in the source experimental studies and considered in the cross-sectional analysis.

Experimental Factor	Code	Description
Chemical treatment	W	Water (irrigation control; no organic supplement)
	EDAR	WWTP-derived organic fertigation matrix (diluted 1/512 *v*/*v*)
	EDAR ST	Sanitized organic WWTP waste
Biological treatment	C0	Non-inoculated control (C0)
	C1	*B. pretiosus* C1-inoculated treatment (~10^8^ CFU·mL^−1^, McFarland 0.5)

Chemical and biological treatments were combined factorially within each source study, generating six treatment groups: WC0, WC1, EDARC0, EDARC1, EDARSTC0, and EDARSTC1.

## Data Availability

*Bacillus pretiosus* C1 was deposited in the CECT and DSMZ bacterial collections under the munbers CECT30674T/DSM 114702T. The Whole genome sequency is deposited in GeneBank under the accession number JAOXJG000000000. Each metagenomic information are deposited in NCBI repository under the accession numbers PRJNA1154929, PRJNA1303565 and PRJNA1153996.

## Supplementary Materials

The following supporting information can be downloaded at https://www.mdpi.com/article/10.3390/life16091558/s1.
