# Supplementary material for: A Bacillus pretiosus Biofertilizer Enhances Early Growth, Nutritional Profile, and Rhizospheric Dynamics in Quercus Species for Forest Restoration
Source: Life (Basel). 2026 Sep 17;16(9):1558. doi: 10.3390/life16091558 (PMC13608531; doi:10.3390/life16091558)
Supplement: Supplementary file 1 [file life-16-01558-s001.zip › life-4505854-supplementary.pdf]

SUPPLEMENTARY MATERIAL

Table S1. Number of biological samples included in each microbiome analysis. Five independent experiments were conducted. Each treatment group was represented by three biological replicates per experiment, resulting in 15 samples per group (N = 30 total samples)

Analysis	Control (n)	Treatment (n)	Total samples (N)
**PCoA**	15	15	30
**PERMANOVA**	15	15	30
**ANOSIM**	15	15	30
**Differential abundance analysis**	15	15	30

Table S2a. Variables included in the cross-sectional analysis and associated functional domain.

2.	**Functional domain**	3.	**Variable Type**	4.	**Variables included**	5.	**Interpretation within the cross-sectional analysis**
**6.**	**Plant-based biometric response**	7.	Growth and development	8.	Shoot weight, shoot length, root length, number of leaves	9.	It allows the effect of *B. pretiosus* on the morphological plant development to be evaluated
**10.**	**Nutritional composition**	11.	Nitrogenous metabolism	12.	Crude protein, soluble protein, available protein, total amino acids	13.	It allows us to assess whether inoculation is associated with changes in the assimilation or accumulation of
**14.**	**Nutritional composition**	15.	Structural Components	16.	Neutral detergent fibre, acid detergent fibre, lignin,	17.	Allows to evaluate possible changes in the structural composition of plant tissue
**18.**	**Nutritional composition**	19.	Carbohydrates and reserves	20.	Starch, soluble sugars, non-structural carbohydrates	21.	Allows the analysis of possible modifications in plant energy metabolism
**22.**	**Nutritional composition**	23.	Mineral profile	24.	P, K, Ca, Mg, and other minerals available in source studies	25.	Allows to assess changes in the mineral nutritional status of plants
**26.**	**Community antibiotic resistance**	27.	Cenoantibiogram	28.	Community MIC values for available antibiotics	29.	It allows to evaluate whether inoculation with *B. pretiosus* is associated with changes in the
**30.**	**Microbial Functional Diversity**	31.	Biolog EcoPlates®	32.	Shannon-Weaver index, AWCD, and/or substrate utilization when available	33.	Allows the evaluation of the global metabolic activity of the rhizospheric community
**34.**	**Microbial taxonomic diversity**	35.	Metagenomics *16S rRNA*	36.	Alpha diversity, beta diversity, relative abundance of bacterial	37.	Allows analyzing changes in the structure and composition of the rhizospheric bacterial community
**38.**	**Functional integration**	39.	Cross-sectional comparison	40.	Direction of the effect of C1 vs. C0 within each matrix	41.	It allows the identification of consistent functional patterns associated with *B. pretiosus*

Table S2b. Integration on the functional profile of *B. pretiosus*.

42. Functional domain	43. Main evidence observed	44. Pattern consistency	45. Functional interpretation
**46. Plant growth**	47. Significant increase in the shoot length in Water and EDAR; positive trend in biomass, especially in EDARST.	48. High for shoot length; moderate for biomass.	49. *B. pretiosus* seems to favor mainly the shoot longitudinal growth, while the effect on biomass accumulation is more matrix-dependent.
**50. Plant nutritional profile**	51. Significant increase in crude protein and soluble protein in EDARST; increase in amino acids in EDAR and EDARST; partial separation in the PCA associated with nitrogenous variables.	52. High for amino acids and proteins; moderate for the overall nutritional profile.	53. *B. pretiosus* seems to favor processes related to plant nitrogen metabolism, promoting the accumulation of nitrogenous compounds.
**54. Carbohydrates and structural components**	55. No significant differences were detected in starch, CSE sugars, lignin, or P between treatments.	56. High for all variables.	57. Inoculation does not appreciably alter the structural components or carbon reserves of the plant.
**58. Community profile of antibiotic resistance**	59. Significant reduction in MIC for IMI and AUG; absence of significant increases for the rest of antibiotics.	60. Registration for IMI and AUG; moderate-high for CTX and moderate for CIP.	61. *B. pretiosus* is not associated with an increased community antibiotic resistance profile and may contribute to maintaining or reducing certain levels of resistance.
**62. Rhizospheric functional diversity**	63. High and comparable values of the Shannon-Weaver index in all treatments; absence of significant differences.	64. Discharge for all treatments.	65. Inoculation maintains the global metabolic activity and functional diversity of the rhizospheric community.
66. **Microbial alpha diversity**	67. The Pielou fairness index did not show significant differences between treatments.	68. High.	69. *B. pretiosus* does not change the richness or overall distribution of microbial diversity.
**70. Beta diversity and community structure**	71. The PCoA showed clustering trends, but PERMANOVA, ANOSIM and PERMDISP did not detect significant differences.	72. Registration for all statistical tests.	73. Inoculation does not cause significant restructuring of the rhizospheric bacterial community.
**74. Abundance of *Bacillus spp.***	75. Relative increase of *Bacillus* spp. in the inoculated samples compared to the controls.	76. High.	77. The strain has the capacity for persistence or detectable colonization in the rhizosphere without massively altering the microbial community.
**78. Global functional profile of the strain**	79. Joint evidence of growth promotion, nutritional improvement, microbiological stability and absence of increased resistance.	80. High, due to the agreement observed between the different functional domains evaluated.	81. *B. pretiosus* has a profile compatible with a PGPB, with positive effects on the plant and little negative impact on the associated microbiota.

Supplementary table S3. Summary of statistical analyses and p-values for the response variables evaluated in the cross-sectional synthesis.

Functional domain	Response variable	Matrix	Comparison	Statistical test	Effect estimates	p-value	Significance
**Plant growth**	Shoot biomass (g FW)	Water	C1 vs C0	ANOVA + post hoc (Tukey)	+0.7	> 0.05	
		EDAR	C1 vs C0	ANOVA + post hoc (Tukey)	+0.9	> 0.05	
		EDARST	C1 vs C0	ANOVA + post hoc (Tukey)	+1.536	0.0739	.
	Shoot length (cm)	Water	C1 vs C0	ANOVA + post hoc (Tukey)	+8.58	0.00583	**
		EDAR	C1 vs C0	ANOVA + post hoc (Tukey)	+14.779	< 0.0001	***
		EDARST	C1 vs C0	ANOVA + post hoc (Tukey)	+1.6	> 0.05	
**Protein nutritional profile**	Soluble protein (g/100 g DM)	Water	C1 vs C0	ANOVA + post hoc (Tukey)	+1.539	0.2970	
		EDAR	C1 vs C0	ANOVA + post hoc (Tukey)	-1.001	0.4968	
		EDARST	C1 vs C0	ANOVA + post hoc (Tukey)	+4.597	0.00238	**
	Crude protein (g/100 g DM)	Water	C1 vs C0	ANOVA + post hoc (Tukey)	+0.7	0.2448	
		EDAR	C1 vs C0	ANOVA + post hoc (Tukey)	+0.984	0.0874	.
		EDARST	C1 vs C0	ANOVA + post hoc (Tukey)	+2.725	< 0.0001	***
	Total amino acids (g/100 g DM)	Water	C1 vs C0	ANOVA + post hoc (Tukey)	+0.799	0.0848	.
		EDAR	C1 vs C0	ANOVA + post hoc (Tukey)	+1.752	0.000255	***
		EDARST	C1 vs C0	ANOVA + post hoc (Tukey)	-1.434	0.00242	**
**Rhizospheric functional diversity**	Shannon–Weaver H′ (144 h)	Water	C1 vs C0	ANOVA + post hoc (Tukey)	-0.23	> 0.05	
		EDAR	C1 vs C0	ANOVA + post hoc (Tukey)	+0.38	> 0.05	
		EDARST	C1 vs C0	ANOVA + post hoc (Tukey)	-0.28	> 0.05	
	Pielou evenness	Overall	C1 vs C0	Kruskal–Wallis	ND	> 0.05	
**Bacterial community – beta diversity**	Weighted UniFrac	Overall	C1 vs C0	PERMANOVA (999 permutations)	F = 7.30; R^2 = 0.777	1	
	Weighted UniFrac	Overall	C1 vs C0	ANOSIM	R = 0.999	1	
	Weighted UniFrac	Overall	C1 vs C0	PERMDISP	F = 1.378	0.248	

	CTX – $\log_2$(MIC)	Overall	C1 vs C0	ANOVA + post hoc (Tukey)	-	0.0812	.
	CIP – $\log_2$(MIC)	Overall	C1 vs C0	ANOVA + post hoc (Tukey)	-	> 0.05	
	AUG – $\log_2$(MIC)	Overall	C1 vs C0	ANOVA + post hoc (Tukey)	-	0.000118	***
		Water	C1 vs C0	ANOVA + post hoc (Tukey)	-1.974	0.00967	**
**Community-level antibiotic susceptibility**		EDAR	C1 vs C0	ANOVA + post hoc (Tukey)	-2.163	0.00761	**
		EDARST	C1 vs C0	ANOVA + post hoc (Tukey)	-1.267	0.0928	.
	IMI – $\log_2$(MIC)	Overall	C1 vs C0	ANOVA + post hoc (Tukey)	-	0.00232	**
		Water	C1 vs C0	ANOVA + post hoc (Tukey)	-2.962	0.00722	**
		EDAR	C1 vs C0	ANOVA + post hoc (Tukey)	-2.600	0.0251	*
		EDARST	C1 vs C0	ANOVA + post hoc (Tukey)	-0.5	> 0.05	

“.” $0.05 \leq p < 0.10$ (trend); * $p < 0.05$; ** $p < 0.01$; *** $p < 0.001$. ND: not determined. “-“: decrease.
